# Supplementary material for: Left-digit bias in out-hospital cardiac arrest: The JCS-ReSS study
Source: PLoS One. 2024 Aug 23;19(8):e0305577. doi: 10.1371/journal.pone.0305577 (PMC11343399; doi:10.1371/journal.pone.0305577)
Supplement: S1 Table — (DOCX) [file pone.0305577.s001.docx]

**Supplemental Table 1. Unadjusted probability of resuscitator behavior by family members, paramedics, and physicians**

| Variable | Cutoff | Coefficient | 95%CI | p-value |
| --- | --- | --- | --- | --- |
| Chest compression (Family members) | 60 | 0.003 | -0.012 – 0.017 | 0.70 |
|  | 70 | 0.004 | -0.013 – 0.026 | 0.51 |
|  | 80 | 0.001 | -0.018 – 0.013 | 0.76 |
|  | 90 | 0.005 | -0.000 – 0.015 | 0.054 |
| Mouth-to mouth ventilation (Family members) | 60 | 0.001 | -0.006 – 0.012 | 0.50 |
|  | 70 | 0.001 | -0.013 – 0.012 | 0.97 |
|  | 80 | -0.002 | -0.006 – 0.001 | 0.20 |
|  | 90 | -0.000 | -0.005 – 0.005 | 0.87 |
| AED usage (Family members) | 60 | 0.001 | -0.002 – 0.004 | 0.45 |
|  | 70 | -0.001 | -0.003 - 0.001 | 0.43 |
|  | 80 | -0.001 | -0.002 – 0.000 | 0.071 |
|  | 90 | -0.000 | -0.001 – 0.001 | 0.95 |
| Chest compression (Paramedics) | 60 | -0.011 | -0.035 – 0.012 | 0.33 |
|  | 70 | -0.013 | -0.048 – 0.013 | 0.26 |
|  | 80 | 0.001 | -0.016 – 0.026 | 0.66 |
|  | 90 | 0.003 | -0.010 – 0.007 | 0.74 |
| Advanced airway management (Paramedics) | 60 | -0.020 | -0.050 – 0.003 | 0.085 |
|  | 70 | -0.006 | -0.021 – 0.003 | 0.13 |
|  | 80 | -0.002 | -0.013 – 0.010 | 0.78 |
|  | 90 | 0.000 | -0.025 – 0.022 | 0.93 |
| AED usage (Paramedics) | 60 | 0.003 | -0.017 – 0.021 | 0.84 |
|  | 70 | -0.002 | -0.034 – 0.038 | 0.91 |
|  | 80 | 0.000 | -0.025 – 0.022 | 0.89 |
|  | 90 | 0.004 | -0.014 – 0.019 | 0.78 |
| ACLS (Physician) | 60 | -0.005 | -0.014 – 0.003 | 0.24 |
|  | 70 | 0.001 | -0.006 – 0.006 | 0.91 |
|  | 80 | 0.001 | -0.011 – 0.015 | 0.75 |
|  | 90 | -0.002 | -0.016 – 0.011 | 0.75 |

Abbreviations: CI, confidence interval
