# Supplementary material for: Left-digit bias in out-hospital cardiac arrest: The JCS-ReSS study
Source: PLoS One. 2024 Aug 23;19(8):e0305577. doi: 10.1371/journal.pone.0305577 (PMC11343399; doi:10.1371/journal.pone.0305577)
Supplement: S2 Table — (DOCX) [file pone.0305577.s002.docx]

**Supplemental Table 2.** Relative risk of probability of primary outcomes in other numeric pairs near the target age threshold

| **Unadjusted** | Threshold  Age, years | Risk Ratio | 95%CI | p-value |
| --- | --- | --- | --- | --- |
| Chest compression (Family members) | 58 vs. 59 | 1.02 | 0.96 – 1.07 | 0.59 |
|  | 60 vs. 61 | 0.98 | 0.93 – 1.03 | 0.49 |
|  | 68 vs. 69 | 1.00 | 0.99 – 1.04 | 0.98 |
|  | 70 vs. 71 | 0.99 | 0.95 – 1.02 | 0.50 |
|  | 78 vs. 79 | 1.08 | 1.00 – 1.17 | 0.04 |
|  | 80 vs. 81 | 1.02 | 0.99 – 1.05 | 0.28 |
|  | 88 vs. 89 | 1.01 | 0.98 – 1.04 | 0.50 |
|  | 90 vs. 91 | 1.00 | 0.97 – 1.03 | 0.93 |
| Mouth-to mouth ventilation (Family members) | 58 vs. 59 | 0.97 | 0.84 – 1.12 | 0.67 |
|  | 60 vs. 61 | 1.02 | 0.89 – 1.17 | 0.76 |
|  | 68 vs. 69 | 0.98 | 0.87 – 1.11 | 0.78 |
|  | 70 vs. 71 | 1.12 | 1.00 – 1.26 | 0.04 |
|  | 78 vs. 79 | 0.99 | 0.91 – 1.09 | 0.88 |
|  | 80 vs. 81 | 1.01 | 0.92 – 1.11 | 0.88 |
|  | 88 vs. 89 | 0.99 | 0.89 – 1.10 | 0.85 |
|  | 90 vs. 91 | 1.00 | 0.89 – 1.12 | 0.97 |
| AED usage (Family members) | 58 vs. 59 | 0.98 | 0.57 – 1.67 | 0.94 |
|  | 60 vs. 61 | 1.21 | 0.73 – 2.01 | 0.46 |
|  | 68 vs. 69 | 0.69 | 0.45 – 1.08 | 0.10 |
|  | 70 vs. 71 | 1.40 | 0.83 – 2.38 | 0.21 |
|  | 78 vs. 79 | 1.10 | 0.68 – 1.80 | 0.70 |
|  | 80 vs. 81 | 0.57 | 0.30 – 1.07 | 0.08 |
|  | 88 vs. 89 | 1.31 | 0.64 – 2.72 | 0.50 |
|  | 90 vs. 91 | 0.63 | 0.28 – 1.43 | 0.27 |
| Chest compression (Paramedics) | 58 vs. 59 | 0.98 | 0.93 – 1.03 | 0.43 |
|  | 60 vs. 61 | 1.03 | 0.99 – 1.08 | 0.15 |
|  | 68 vs. 69 | 1.01 | 0.97 – 1.03 | 0.76 |
|  | 70 vs. 71 | 1.00 | 0.97 – 1.03 | 0.92 |
|  | 78 vs. 79 | 0.98 | 0.96 – 1.01 | 0.16 |
|  | 80 vs. 81 | 0.98 | 0.96 – 1.01 | 0.14 |
|  | 88 vs. 89 | 0.99 | 0.96 – 1.02 | 0.45 |
|  | 90 vs. 91 | 1.00 | 0.97 – 1.03 | 0.88 |
| Advanced airway management (Paramedics) | 58 vs. 59 | 0.94 | 0.89 – 0.998 | 0.043 |
|  | 60 vs. 61 | 1.02 | 0.96 – 1.08 | 0.51 |
|  | 68 vs. 69 | 1.00 | 0.96 – 1.04 | 0.88 |
|  | 70 vs. 71 | 1.01 | 0.98 – 1.05 | 0.48 |
|  | 78 vs. 79 | 1.01 | 0.98 – 1.04 | 0.40 |
|  | 80 vs. 81 | 0.97 | 0.94 – 0.997 | 0.03 |
|  | 88 vs. 89 | 0.97 | 0.94 – 1.00 | 0.09 |
|  | 90 vs. 91 | 0.97 | 0.93 – 1.01 | 0.12 |
| AED usage (Paramedics) | 58 vs. 59 | 0.98 | 0.91 – 1.05 | 0.51 |
|  | 60 vs. 61 | 1.00 | 0.93 – 1.07 | 0.97 |
|  | 68 vs. 69 | 0.97 | 0.91 – 1.03 | 0.33 |
|  | 70 vs. 71 | 0.97 | 0.91 – 1.03 | 0.31 |
|  | 78 vs. 79 | 0.94 | 0.89 – 1.002 | 0.06 |
|  | 80 vs. 81 | 0.98 | 0.92 – 1.04 | 0.52 |
|  | 88 vs. 89 | 1.01 | 0.92 – 1.11 | 0.81 |
|  | 90 vs. 91 | 0.96 | 0.87 – 1.07 | 0.49 |
| ACLS (Physician) | 58 vs. 59 | 1.00 | 0.98 – 1.02 | 0.91 |
|  | 60 vs. 61 | 1.01 | 0.99 – 1.02 | 0.28 |
|  | 68 vs. 69 | 1.00 | 0.99 – 1.01 | 0.60 |
|  | 70 vs. 71 | 1.00 | 0.99 – 1.01 | 0.62 |
|  | 78 vs. 79 | 1.01 | 1.0004 – 1.02 | 0.041 |
|  | 80 vs. 81 | 1.00 | 0.99 – 1.01 | 0.71 |
|  | 88 vs. 89 | 1.01 | 1.00 – 1.02 | 0.16 |
|  | 90 vs. 91 | 1.00 | 0.99 – 1.01 | 0.45 |
| **Adjusted** | Threshold  Age, years | Risk Ratio | 95%CI | p-value |
| Chest compression (Family members) | 58 vs. 59 | 1.03 | 0.92 – 1.14 | 0.55 |
|  | 60 vs. 61 | 0.97 | 0.89 – 1.06 | 0.53 |
|  | 68 vs. 69 | 1.00 | 0.94 – 1.07 | 0.97 |
|  | 70 vs. 71 | 0.98 | 0.92 – 1.04 | 0.54 |
|  | 78 vs. 79 | 1.03 | 0.98 – 1.08 | 0.29 |
|  | 80 vs. 81 | 1.03 | 0.98 – 1.08 | 0.21 |
|  | 88 vs. 89 | 1.02 | 0.96 – 1.07 | 0.57 |
|  | 90 vs. 91 | 1.00 | 0.94 – 1.06 | 0.94 |
| Mouth-to mouth ventilation (Family members) | 58 vs. 59 | 0.97 | 0.82 – 1.14 | 0.69 |
|  | 60 vs. 61 | 1.03 | 0.88 – 1.19 | 0.74 |
|  | 68 vs. 69 | 0.98 | 0.86 – 1.12 | 0.79 |
|  | 70 vs. 71 | 1.14 | 1.01 – 1.28 | 0.04 |
|  | 78 vs. 79 | 0.99 | 0.90 – 1.10 | 0.87 |
|  | 80 vs. 81 | 1.01 | 0.91 – 1.11 | 0.86 |
|  | 88 vs. 89 | 0.99 | 0.88 – 1.10 | 0.79 |
|  | 90 vs. 91 | 1.00 | 0.88 – 1.13 | 0.94 |
| AED usage (Family members) | 58 vs. 59 | 0.98 | 0.57 – 1.69 | 0.95 |
|  | 60 vs. 61 | 1.22 | 0.73 – 2.04 | 0.44 |
|  | 68 vs. 69 | 0.69 | 0.44 – 1.07 | 0.10 |
|  | 70 vs. 71 | 1.40 | 0.82 – 2.39 | 0.21 |
|  | 78 vs. 79 | 1.10 | 0.68 – 1.80 | 0.69 |
|  | 80 vs. 81 | 0.57 | 0.30 – 1.08 | 0.08 |
|  | 88 vs. 89 | 1.29 | 0.67 – 2.52 | 0.45 |
|  | 90 vs. 91 | 0.64 | 0.28 – 1.44 | 0.28 |
| Chest compression (Paramedics) | 58 vs. 59 | 0.96 | 0.86 – 1.06 | 0.40 |
|  | 60 vs. 61 | 1.07 | 0.97 – 1.18 | 0.16 |
|  | 68 vs. 69 | 1.01 | 0.94 – 1.08 | 0.79 |
|  | 70 vs. 71 | 1.00 | 0.93 – 1.06 | 0.91 |
|  | 78 vs. 79 | 0.96 | 0.92 – 1.02 | 0.16 |
|  | 80 vs. 81 | 0.96 | 0.91 – 1.01 | 0.14 |
|  | 88 vs. 89 | 0.98 | 0.92 – 1.04 | 0.47 |
|  | 90 vs. 91 | 1.00 | 0.93 – 1.06 | 0.88 |
| Advanced airway management (Paramedics) | 58 vs. 59 | 0.90 | 0.82 –0.997 | 0.043 |
|  | 60 vs. 61 | 1.03 | 0.94 – 1.13 | 0.49 |
|  | 68 vs. 69 | 1.01 | 0.94 – 1.07 | 0.87 |
|  | 70 vs. 71 | 1.02 | 0.96 – 1.09 | 0.46 |
|  | 78 vs. 79 | 1.02 | 0.97 – 1.08 | 0.36 |
|  | 80 vs. 81 | 0.95 | 0.90 – 0.997 | 0.039 |
|  | 88 vs. 89 | 0.96 | 0.90 – 1.01 | 0.11 |
|  | 90 vs. 91 | 0.95 | 0.89 – 1.01 | 0.12 |
| AED usage (Paramedics) | 58 vs. 59 | 0.97 | 0.81 – 1.16 | 0.77 |
|  | 60 vs. 61 | 1.08 | 0.92 – 1.27 | 0.35 |
|  | 68 vs. 69 | 0.96 | 0.85 – 1.08 | 0.48 |
|  | 70 vs. 71 | 0.96 | 0.85 – 1.08 | 0.48 |
|  | 78 vs. 79 | 0.94 | 0.85 – 1.04 | 0.21 |
|  | 80 vs. 81 | 1.05 | 0.95 – 1.16 | 0.38 |
|  | 88 vs. 89 | 1.05 | 0.93 – 1.19 | 0.44 |
|  | 90 vs. 91 | 0.93 | 0.80 – 1.07 | 0.30 |
| ACLS (Physician) | 58 vs. 59 | 1.01 | 0.87 – 1.16 | 0.91 |
|  | 60 vs. 61 | 0.93 | 0.81 – 1.06 | 0.28 |
|  | 68 vs. 69 | 1.03 | 0.93 – 1.14 | 0.59 |
|  | 70 vs. 71 | 0.98 | 0.89 – 1.07 | 0.62 |
|  | 78 vs. 79 | 0.92 | 0.86 – 0.997 | 0.042 |
|  | 80 vs. 81 | 0.99 | 0.91 – 1.06 | 0.72 |
|  | 88 vs. 89 | 0.94 | 0.86 – 1.02 | 0.16 |
|  | 90 vs. 91 | 0.96 | 0.87 – 1.06 | 0.43 |
